# Tuberculin Skin Test and Boosted Reactions among Newly Employed Healthcare Workers: An Observational Study

**DOI:** 10.1371/journal.pone.0064563

**Published:** 2013-05-24

**Authors:** Song Yee Kim, Moo Suk Park, Young Sam Kim, Se Kyu Kim, Joon Chang, Dongeun Yong, Hyun Sook Kim, Kyungwon Lee, Young Ae Kang

**Affiliations:** 1 Division of Pulmonology, Department of Internal Medicine, Yonsei University College of Medicine, Seoul, Republic of Korea; 2 Department of Laboratory Medicine, Yonsei University College of Medicine, Seoul, Republic of Korea; McGill University, Canada

## Abstract

**Objective:**

To evaluate the prevalence of and factors associated with latent tuberculosis infection (LTBI) based on the tuberculin skin test (TST) and to estimate the boosted reaction rate among newly employed healthcare workers (HCWs).

**Design:**

Newly employed HCWs between January 2010 and July 2012 at Severance Hospital in South Korea were enrolled in this study. A one-step TST was conducted before October 2011, and a two-step TST after October 2011.

**Results:**

Of 2132 participants, 778 (36.5%) had positive TST results. Being older (odds ratio [OR] 1.10, 95% confidence interval [CI] 1.06–1.13, *P*<0.001), male (OR 1.78, 95% CI 1.21–2.62, *P* = 0.003), rejoining the hospital workforce (OR 1.58, 95% CI 1.04–2.40, *P* = 0.032), and having a previous history of tuberculosis (TB) (OR 18.21, 95% CI 2.15–154.10, *P* = 0.008) during the one-step period, and being older (OR 1.15, 95% CI 1.10–1.21, *P*<0.001) during the two-step period were significantly associated with a positive TST. A two-step TST was performed in 556 HCWs, and a boosted reaction was observed in 79 (14.2%). The induration size on the first TST (5–9-mm group) was the only factor associated with a boosted reaction on the second TST.

**Conclusions:**

The prevalence of LTBI based on the TST among newly employed HCWs was high. The boosted reaction rate on two-step TST was not low; therefore, the use of two-step TST may be necessary for regular monitoring in countries with an intermediate TB burden and a high rate of Bacillus Calmette-Guérin vaccination.

## Introduction

It has long been recognized that healthcare workers (HCWs) are at high risk of a *Mycobacterium tuberculosis (M. TB)* infection due to occupational exposure [1], [2]. To control tuberculosis (TB) among HCWs, routine screening and appropriate treatment for latent TB infections (LTBIs) are recommended [3]. For regular monitoring, it is important to have baseline data regarding LTBIs in HCWs. However, limited information about LTBIs among newly employed HCWs is available.

Although the interferon-γ release assay (IGRA) was recently developed, the tuberculin skin test (TST) is still widely used to diagnose LTBIs. However, the test has several problems in terms of usage and interpretation. In particular, a boosted reaction may occur on repeated testing, which can cause misinterpretation as a conversion of the TST [4]. A boosted reaction is regarded as correlated with the initial TST reaction and Bacillus Calmette-Guérin (BCG) vaccination [1], [4]–[6]. Therefore, because repeated testing is needed for monitoring, an evaluation of boosted reactions is important among HCWs in areas with a high TB burden and where BCG vaccination is mandatory.

South Korea has an intermediate incidence of TB (85–110/100 000 population per year) [7] and BCG vaccination is given at birth to most infants. Recent Korean guidelines have recommended regular checkups for TB infection among HCWs at high risk for exposure [8]. However, limited data are available regarding LTBIs among newly employed HCWs in South Korea.

The present study was performed to evaluate the prevalence of and associated factors for LTBIs based on the TST, and to estimate the boosted reaction rate among newly employed HCWs at a tertiary referral hospital in South Korea.

## Patients and Methods

### Ethics Statement

The research protocol was approved by the Institutional Review Board (IRB) of Severance Hospital. The requirement for informed consent was waived by the IRB due to the retrospective nature of the analysis (IRB No. 4-2012-0780).

### Study Design and Participants

This study was conducted at Severance Hospital (Seoul, Republic of Korea), a tertiary referral hospital with approximately 2000 beds. About 600 patients with smear- or culture-positive TB are managed at this hospital each year. The participants were HCWs who were newly employed between January 2010 and July 2012. A total of 2160 HCWs were employed during the study period. Because 28 HCWs during the study period were not screened due to pregnancy or administrative omission, 2132 HCWs were enrolled in this study. Each individual underwent a TST with a simple chest radiograph and was examined for comorbidities, previous TB history, occupational category (doctors, nurses, technicians, and others), and previous occupational history as an HCW according to hospital policy. Technicians were defined as technical employees performing services in radiology, laboratory, pathology, and physiotherapy. HCWs who rarely came into contact with patients or who worked in the medical school were defined as others. Previous employment history considered only employment at Severance Hospital; this was expressed as rejoining.

All participants, except retired HCWs, were followed up until September 2012.

### Tuberculin Skin Test

A TST was performed on the forearm in accordance with the Mantoux method [4] using a 2-TU dose of the purified protein derivative RT 23 (Statens Serum Institute, Copenhagen, Denmark). Transverse induration was measured in mm between 48 and 72 h after injection.

A one-step TST was conducted before October 2011; a two-step TST was conducted after October 2011 because the hospital policy changed in October 2011. A total of 1171 HCWs were enrolled during the one-step period, and 961 HCWs were enrolled during the two-step period. The two-step TST was performed as follows: the TST was repeated 1 to 3 weeks later in subjects with negative initial TST results. A positive TST was defined as an induration of ≥10 mm in diameter [4]. A boosted reaction was defined as induration size of <10 mm on the first TST and an induration size of ≥10 mm on the second TST.

### Statistical Analysis

All data are shown as numbers (percentages) or medians and interquartile ranges (IQRs). Pearson’s Chi-squared test or Fisher’s exact test was used to compare categorical variables, and the Mann-Whitney U-test was used to compare continuous variables. A logistic regression analysis was performed for multivariate analysis. The data were analyzed using SPSS (v. 18.0; SPSS Inc., Chicago, IL). In all analyses, P<0.05 (two-tailed) was taken to indicate statistical significance. Odds ratios (ORs) and their accompanying 95% confidence intervals (CIs) were estimated from the multivariate logistic regression model. In terms of age, OR was increased per year of age.

## Results

### Participant Characteristics

The baseline characteristics of the participants according to the period are presented in Table 1. The median age of the HCWs was 27 years (range, 20–65 years), and 1407 (66.0%) of the participants were women. The study population consisted of 650 (30.5%) doctors, 571 (26.8%) nurses, and 376 (17.6%) technicians. Two hundred and seven (9.7%) were rejoining HCWs. Twenty-four (1.1%) had a previous history of TB. Simple chest radiographs showed that 103 (4.8%) of the subjects had previously healed TB, 3 (0.1%) had active TB, and the remaining 2018 (94.7%) had normal findings. The proportion of subjects rejoining the hospital workforce and with a previous TB history was higher and the proportion of male subjects was lower during the one-step period than during the two-step period.

**Table 1:** Baseline characteristics of participants.

Characteristics	Period		Total (n = 2132)	*P*-value
	One-step period (n = 1171)	Two-step period (n = 961)		
Age (yrs), median (range)	27 (21–65)	27 (20–54)	27 (20–65)	0.273
20–29	774 (66.1)	682 (71.0)	1456 (68.3)	
30–39	357 (30.5)	258 (26.8)	615 (28.8)	
40–49	36 (3.1)	20 (2.1)	56 (2.6)	
50–59	2 (0.2)	1 (0.1)	3 (0.1)	
60–69	2 (0.2)	0 (0)	2 (0.1)	
Gender; male, *n* (%)	369 (31.5)	356 (37.0)	725 (34.0)	0.008
BMI (median, IQR)	21.0 (19.3–22.9)	20.5 (19.1–22.5)	20.8 (19.2–22.7)	0.011
Rejoined	146 (12.5)	61 (6.3)	207 (9.7)	<0.001
Previous TB history	20 (1.7)	4 (0.4)	24 (1.1)	0.006
Findings on CXR*				0.228
Normal	1109 (94.7)	909 (94.6)	2018 (94.7)	
Previously healed TB	53 (4.5)	50 (5.2)	103 (4.8)	
Active pulmonary TB	3 (0.3)	0 (0)	3 (0.1)	
Job category				<0.001
Doctor	287 (24.5)	363 (37.8)	650 (30.5)	
Nurse	376 (32.1)	195 (20.3)	571 (26.8)	
Technician$	237 (20.2)	139 (14.5)	376 (17.6)	
Others	271 (23.1)	264 (27.5)	535 (25.1)	
TST induration(median, IQR), mm	5.0 (0.0–12.0)	6.0 (0.0–12.0)	5.0 (0.0–12.0)	0.046
TST ≥5 mm	589 (50.3)	421 (43.8)	1129 (53.0)	0.007
TST ≥10 mm	392 (33.5)	386 (40.2)	778 (36.5)	0.002

BMI, body mass index; IQR, interquartile range; TB, tuberculosis; CXR, simple chest radiography; TST, tuberculin skin test.

* Data could not be obtained for eight participants.

$ Technicians were defined as technical employees performing radiologic, laboratory, pathologic, and physiotherapeutic services.

### TST Results and Associated Factors

**Figure 1:**
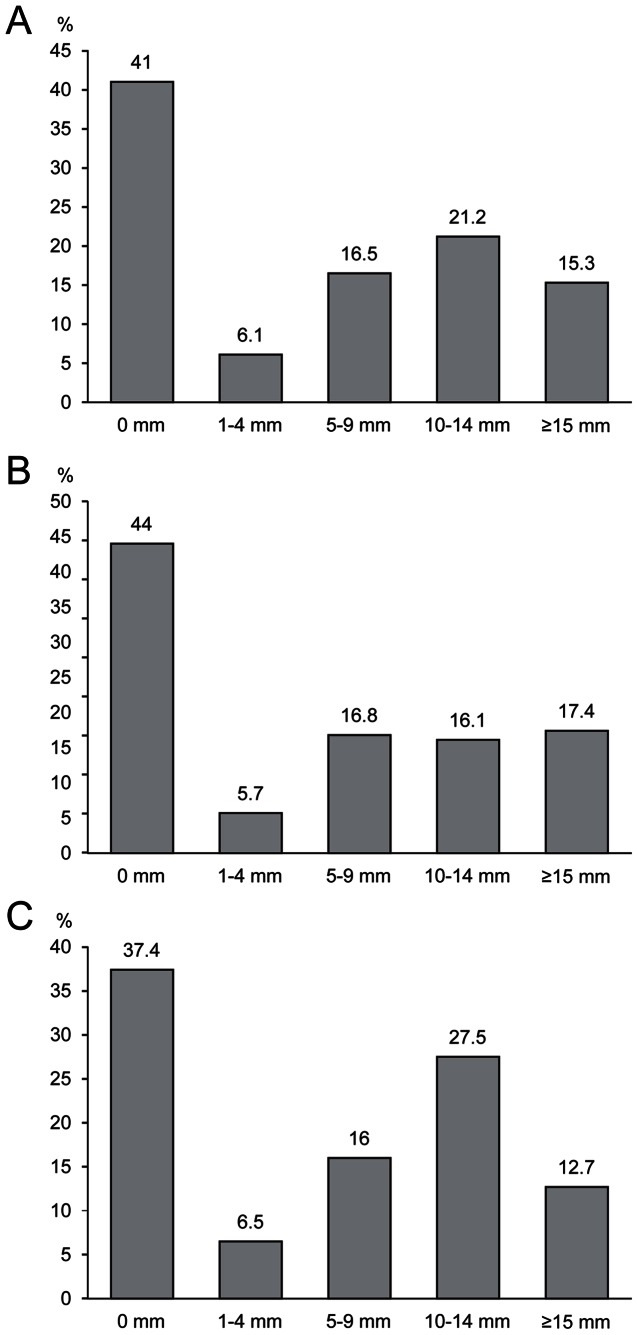
Distribution of tuberculin skin test reactions. A. Overall. B. One-step period. C. Two-step period.

**Figure 2:**
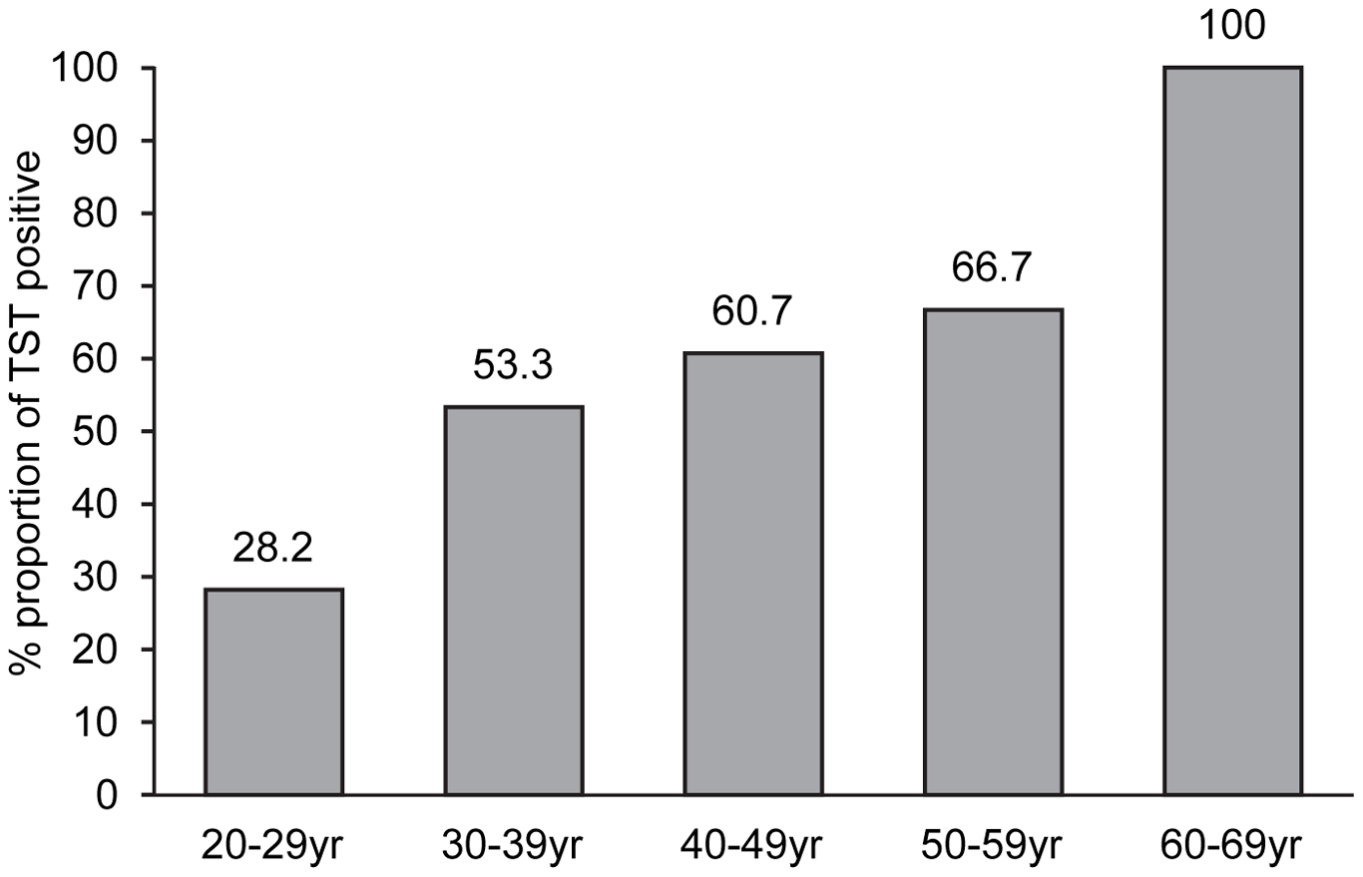
Trend of TST positivity according to age.

The proportion of positive TST was 36.5% overall. The prevalence of TST positivity was 33.5%, and the median TST induration size was 5.0 mm (IQR, 0.0–12.0 mm) during the one-step period. During the two-step period, the prevalence of TST positivity was 40.2%, and the median TST induration size was 6.0 mm (IQR, 0.0–12.0 mm). Figure 1 shows the distribution of TST reactions in each period. Eight hundred and four (41%) subjects had no measurable indurations, and the other distribution was concentrated around 10 mm. Tables 2 and 3 present the factors associated with TST positivity as determined by univariate and multivariate analyses according to the period. Finally, being older (OR 1.10, 95% CI 1.06–1.13, *P*<0.001), male (OR 1.78, 95% CI 1.21–2.62, *P* = 0.003), rejoining the hospital workforce (OR 1.58, 95% CI 1.04–2.40, *P* = 0.032), and having a previous history of TB (OR 18.21, 95% CI 2.15–154.10, *P* = 0.008) were significantly associated with positive TST results by multivariate analysis during the one-step period. Being older (OR 1.15, 95% CI 1.10–1.21, *P*<0.001) was significantly associated with positive TST results by multivariate analysis during the two-step period. The trend of TST positivity according to age is presented in Figure 2. Job category and the presence of previously healed TB on simple chest radiography showed no association on multivariate analysis.

**Table 2:** Clinical factors associated with a positive TST by univariate and multivariate analyses during the one-step period.

		TST		Univariate analysis				Multivariate analysis		
Clinical factor		Negative (n = 779)	Positive (n = 392)	OR		95% CI	*P*	OR	95% CI	*P*
Age (yrs), median (range)		25 (21–53)	29 (21–65)	1.12		1.10–1.15	<0.001	1.10	1.06–1.13	<0.001
Gender; male, *n* (%)		200 (25.7)	169 (43.1)	2.19		1.70–2.84	<0.001	1.78	1.21–2.62	0.003
BMI (median, IQR)		20.8 (19.1–22.6)	21.5 (19.7–23.3)	1.07		1.02–1.13	0.004	0.99	0.93–1.05	0.726
Rejoined		77 (9.9)	69 (17.6)	1.95		1.37–2.77	<0.001	1.58	1.04–2.40	0.032
Previous TB history		2 (0.3)	18 (4.6)	18.70		4.32–81.00	<0.001	18.21	2.15–154.10	0.008
Finding of CXRs*										
Normal	751 (96.9)		358 (91.8)	1.00				1.00		
Previously healed TB	24 (3.1)		29 (7.4)	2.54	1.46–4.42		0.001	1.15	0.55–2.43	0.711
Active pulmonary TB	0 (0.0)		3 (0.8)							
Job category										
Doctor	154 (19.8)		133 (33.9)	1.55	1.10–2.18		0.012	1.05	0.69–1.60	0.821
Nurse	273 (35.0)		103 (26.3)	0.68	0.49–0.95		0.023	1.26	0.81–1.97	0.313
Technician^$^	178 (22.8)		59 (15.1)	0.60	0.41–0.87		0.008	0.82	0.52–1.31	0.408
Others	174 (22.3)		97 (24.7)	1.00				1.00		

TST, tuberculin skin test; BMI, body mass index; IQR, interquartile range; TB, tuberculosis; CXR, simple chest radiography; OR, odds ratio; CI, confidence interval.

* Data could not be obtained for six participants.

$ Technicians are defined as technical employees performing radiologic, laboratory, pathologic, and physiotherapeutic services.

**Table 3:** Clinical factors associated with a positive TST through univariate and multivariate analyses during the two-step period.

Clinical factor	TST		Univariate analysis			Multivariate analysis		
	Negative (n = 575)	Positive (n = 386)	OR	95% CI	*P*	OR	95% CI	*P*
Age (yrs), median (range)	25 (21–47)	29 (20–54)	1.16	1.13–1.20	<0.001	1.15	1.10–1.21	<0.001
Gender; male, *n* (%)	191 (33.2)	165 (42.7)	1.50	1.15–1.96	0.003	1.21	0.78–1.87	0.405
BMI (median, IQR)	20.2 (18.9–22.3)	20.9 (19.5–22.8)	1.09	1.03–1.15	0.005	1.02	0.95–1.10	0.541
Rejoined	26 (4.5)	35 (9.1)	2.11	1.25–3.56	0.005	0.90	0.46–1.77	0.769
Previous TB history	0 (0.0)	4 (1.0)	–			–		
Findings of CXRs*								
Normal	551 (96.2)	358 (92.7)	1.00			1.00		
Previously healed TB	22 (3.8)	28 (7.3)	1.96	1.10–3.48	0.022	1.66	0.78–3.52	0.187
Active pulmonary TB	0 (0.0)	0 (0.0)						
Job category								
Doctor	205 (35.7)	158 (40.9)	1.08	0.78–1.49	0.642			
Nurse	125 (21.7)	70 (18.1)	0.78	0.54–1.15	0.211			
Technician$	91 (15.8)	48 (12.4)	0.74	0.48–1.13	0.164			
Others	154 (26.8)	110 (28.5)	1.00					

TST, tuberculin skin test; BMI, body mass index; IQR, interquartile range; TB, tuberculosis; CXR, simple chest radiography; OR, odds ratio; CI, confidence interval.

* Data could not be obtained for two participants.

$ Technicians were defined as technical employees performing radiologic, laboratory, pathologic, and physiotherapeutic services.

### Boosted Reaction and Associated Factors

A two-step TST was performed in 556 HCWs, and a boosted reaction was observed in 79 (14.2%). Older or male subjects, or subjects with previously healed TB on chest radiography or a higher induration size on the first TST (especially in the 5–9-mm group) tended to show a boosted reaction on the TST. By multivariate analysis, induration size on the first TST (5–9-mm group) was the only factor associated with a boosted reaction on the second TST (Table 4).

**Table 4:** Clinical factors associated with a boosted TST reaction.

Characteristics	Boosted reaction		OR (95% CI)	*P*
	Absence	Presence		
Subjects	477 (85.8)	79 (14.2)		
Age (yrs)	25 (21–47)	26 (20–54)		
20–24	170 (35.6)	17 (21.5)	1.00	
25–29	218 (45.7)	38 (48.1)	1.63 (0.72–3.70)	0.244
30–34	66 (13.8)	16 (20.3)	2.30 (0.85–6.26)	0.103
35–39	14 (2.9)	6 (7.6)	1.63 (0.22–11.98)	0.630
>40	9 (1.9)	2 (2.5)	–	0.99
Gender; male, *n* (%)	135 (28.3)	33 (41.8)	1.47 (0.63–3.45)	0.372
BMI (median, IQR)	20.0 (18.9–22.1)	21.7 (19.8–22.7)	1.16 (0.99–1.36)	0.060
Rejoined	19 (4.0)	3 (3.8)	0.23 (0.02–2.26)	0.207
Previous TB history	0 (0)	0 (0)	–	–
Previously healed TB on CXR*	18 (3.8)	6 (7.6)	1.65 (0.40–6.74)	0.485
TST induration (median, IQR), mm	0 (0–3)	6 (0–8)		
0 mm	331 (69.4)	25 (31.6)	1.00	
1–4 mm	45 (9.4)	6 (7.6)	2.53 (0.83–7.75)	0.103
5–9 mm	101 (21.2)	48 (60.8)	6.51 (3.13–13.53)	<0.001
Job category				
Doctor	129 (27.0)	17 (21.5)		
Nurse	121 (25.4)	17 (21.5)		
Technician$	90 (18.9)	14 (17.7)		
Others	137 (28.7)	31 (39.2)		
Interval between 1^st^ and 2^nd^ TST days(median, range)	9 (4–65)	10 (7–35)		

TST, tuberculin skin test; OR, odds ratio; CI, confidence interval; BMI, body mass index; IQR, interquartile range; TB, tuberculosis; CXR, simple chest radiography.

* Data could not be obtained for one participant.

$ Technicians were defined as technical employees performing radiologic, laboratory, pathologic, and physiotherapeutic services.

### Follow-up

The median length of follow-up was 9.9 months (range, 3.5–30.9 months). Active TB developed in two HCWs during the follow-up period. Both subjects were women, 27 and 30 years old, respectively. One was a nurse and the other was a doctor, and the doctor had rejoined the hospital. Simple chest radiography at employment showed that one had previously healed TB and the other had normal finding. The results of a TST at employment were positive in two subjects (induration sizes, 15 and 24 mm).

## Discussion

In the present study, the prevalence of an LTBI based on a TST among newly employed HCWs was 36.5%, and factors associated with TST positivity were older age, male, rejoining the hospital workforce, and a previous history of TB. In addition, we showed that the boosted reaction rate on a two-step TST was not low (14.2%), and that a boosted reaction was related to the induration size on the first TST (5–9-mm group).

The reported TST positivity among HCWs can vary according to the characteristics of the participants. TST positivity in this study was similar to that in a previous study performed at our institution (34% among subjects at low risk of exposure to TB) [9]. The reported rates of TST positivity among newly employed HCWs at other institutions in South Korea have been higher (51.5%) and lower (26%) than ours [10], [11]. This discrepancy may be due to the different characteristics of HCWs or setting. One previous study that reported higher TST positivity included a relatively high proportion of HCWs with exposure to TB and prior anti-TB treatment [11]. In contrast, lower positivity was reported in the setting of a one-step TST [10]. Considering these differences in study design and setting, the rate of LTBIs based on the TST in our study was relatively considerable.

There is presently no gold standard diagnostic test for LTBI. Nevertheless, our study showed that being older, male, rejoining the hospital workforce, and having a previous history of TB were associated with TST positivity, although there were slight differences according to the period due to the small discrepancy in baseline characteristics during the one-step and two-step periods. Cristopoulos *et al.* and Altunoren *et al.* reported that older age is a risk factor for TB [12], [13]. Jimenez-Corona *et al.* reported that there may be sex-related differences in incidence rates of TB in the general population due to the dynamics of local spread or the degree of environmental exposure [14]. Rejoining the hospital workforce was a reasonable associated factor for TST positivity because occupational exposure is a known risk factor for *M. TB* infection [1], [2]. Previous TB history was also considered a risk factor for an LTBI in other studies [15], [16]. Taken together, our data suggest that the TST may reflect the risk of LTBI among HCWs, and the observation that the HCWs who developed active TB after employment had a positive TST at employment supports this hypothesis.

Recently, the IGRAs, QuantiFERON-TB Gold In-Tube test (Cellestis Ltd., Carnegie, Victoria, Australia), and T.SPOT TB test (Oxford Immunotec, Abingdon, UK) have been developed and used in clinical practice, and many studies have suggested that IGRAs could overcome the limitations of the TST. However, serial IGRAs can reveal variations and serial monitoring in HCWs by IGRAs is challenging [17], [18]; thus, the TST is still useful despite the possibility of a boosted reaction, particularly in a BCG-vaccinated population [4]–[6], [19].

The boosted reaction rate for the two-step TST varies according to the characteristics of the study population (e.g., age distribution, previous exposure to TB or nontuberculous mycobacteria, and BCG vaccination status) [20]–[24]. The boosted reaction rate (14.2%) in our study was intermediate between those of populations in which BCG vaccination was given at infancy (8%) and at 5 years or older (18%) [19]. In South Korea until 1997, the BCG vaccine was given at birth and again at the age of 12 or 13 years if the child proved to be a TST non-responder. BCG revaccination was stopped in 1997 according to the recommendation of the WHO in 1995 due to the lack of evidence [25]. Considering the ages of the participants, the HCWs in our study would have consisted of a mixture of those vaccinated with BCG once and twice. Therefore, the observed boosted reaction rate among newly employed HCWs in this study is reasonable and not low compared to the general population.

The observation that induration size on the first TST, especially 5–9 mm, was the only factor associated with a boosted reaction is consistent with previous reports [26], [27]. Based on the observed rate of a boosted reaction and associated factors, the two-step TST may be useful in newly employed HCWs for regular TST monitoring, and interpretation of the TST should be performed with caution in some groups. However, a substantial number of boosted reactions developed in HCWs with a 0-mm induration size at the first TST. This result indicates that the two-step TST cannot be performed on only a selected group and should be performed on all individuals without a prior skin test in the 12-month period before beginning employment, as recommended by the US Centers for Disease Control and Prevention (CDC) TB infection control guidelines [3].

In our hospital, the Department of Infectious Disease Control manages the infection control policy. Rooms for infectious patients with TB and the area for high-risk procedures such as bronchoscopy are isolated and equipped with negative-pressure ventilation. Patients who are confirmed to have infectious TB or are suspicious for infectious TB are immediately isolated. HCWs or visitors entering such rooms should wear N95 respirators.

In addition, we have a policy for monitoring TB infection in HCWs. We perform TSTs and chest radiographs in newly employed HCWs and repeatedly perform TSTs and chest radiographs in HCWs who are exposed to patients with TB. We also annually perform TSTs and chest radiographs in HCWs who work in departments related to TB. However, because there is no consensus regarding the optimal screening strategy for LTBI in HCWs in Korea, this study will provide baseline data.

Our study has several limitations. First, this study was conducted at a single institution. Second, we could not obtain data regarding the history of BCG vaccination. However, a high rate of BCG vaccination can be expected in our participants because BCG vaccination is mandatory in South Korea and previous data indicate that the prevalence of BCG scars in Korean infants is about 88% [28]. Third, there was no information about TB exposure before employment. Fourth, we had no information about previous occupational history as an HCW except at our institution. Occupational exposure is an important risk factor for TB infection in HCWs, and information about employment history is necessary to accurately evaluate the risk factors for an LTBI. Fifth, the mixed one- and two-step TST group was a limitation in evaluating the prevalence and risk factors for having a positive test. The prevalence of LTBI could have been underestimated during the one-step period, and the boosted reaction could have been due to a combination of LTBI and prior BCG vaccination. This mixture was due to the change in the hospital policy during the study period.

### Conclusions

The prevalence of LTBI based on the TST among newly employed HCWs was high. The boosted reaction rate on the two-step TST was not low; therefore, the use of the two-step TST may be necessary for regular monitoring in countries with an intermediate TB burden and a high rate of BCG vaccination.
